# Plasticity of the HEK-293 cells, related to the culture media, as platform to produce a subunit vaccine against classical swine fever virus

**DOI:** 10.1186/s13568-019-0864-8

**Published:** 2019-09-05

**Authors:** Elianet Lorenzo, Lidice Méndez, Elsa Rodríguez, Nemecio Gonzalez, Gleysin Cabrera, Carlos Pérez, Rafael Pimentel, Yusmel Sordo, Maria P. Molto, Talia Sardina, Alina Rodríguez-Mallon, Mario P. Estrada

**Affiliations:** 1 Animal Biotechnology Division, Center for Genetic Engineering and Biotechnology, Playa, P.O. Box 6162, Zip Code: 10600 Havana, Cuba; 2 Development Division, Center for Genetic Engineering and Biotechnology, 70100 Camagüey, Cuba; 3 Glicobiology Division, Center for Genetic Engineering and Biotechnology, 10600 Havana, Cuba

**Keywords:** Glycoprotein E2-CD154, HEK-293 cells, Culture media, Growth profiles, N-Glycosylation

## Abstract

Classical swine fever (CSF) is a contagious disease that causes a high mortality to domestic and wild pigs. Its causative agent is an enveloped *Pestivirus* named Classical Swine Fever Virus (CSFV). Due to the huge economic affectations produced by this disease to porcine industry, several vaccines have been developed using principally the CSFV E2 glycoprotein. Recently, a subunit vaccine based on this structural protein of the CSFV fused to the porcine CD154 molecule as immunomodulator named E2-CD154 was assayed by us. This chimeric protein was produced in the Human Embryonic Kidney (HEK-293) cell line. In this work, the growth and the expression profiles of HEK-293 E2-CD154 cells in four commercially available culture media were studied. The oligosaccharide structures in the N-glycosylation patterns of the E2-CD154 protein produced by this cell line in 10 L fermenters with two different culture media were also analyzed. In addition, the neutralizing antibody response generated in mice vaccinated with these antigens was assayed. Our results suggest that the culture media CDM4HEK293 and SFM4HEK293 which are recommended for HEK-293 growth are the best choice to growth the cell clone expressing the E2-CD154 protein. The glycosylation pattern and the neutralizing antibody response generated by the E2-CD154 protein were independent of the culture medium used which demonstrates the high reproducibility and consistency among protein batches produced by HEK-293 cells even in different culture conditions.

## Introduction

Classical swine fever (CSF) is a contagious and often fatal disease that affects both domestic and wild pigs. The causative agent is an enveloped virus with a genome of positive polarity single strand RNA. It belongs to the genus *Pestivirus* of the *Flaviviridae* family and its name is generic of the disease that it produces (CSFV) (Moennig 2000; Murphy et al. 1995). CSF is distributed worldwide and causes great losses in those countries where the pig industry is an important economic sector. For several years, multiple vaccine candidates against CSFV based mainly on viral E2 glycoprotein (subunit vaccines) have been evaluated (Hulst et al. 1993). This viral protein is considered the most immunogenic due to its ability to induce a response of neutralizing antibodies against the virus (Uttenthal et al. 2001; van Zijl et al. 1991; Ziegler and Kaden 2001).

There have been several attempts to produce the E2 protein as a vaccine antigen in different expression systems. However, the E2 protein produced in baculovirus/insect cell system is not capable to confer sterilizing immunity (Bouma et al. 1999). Moreover, this production system is expensive, technically demanding and there have been problems with the secretion and post-translational processing when recombinant glycoproteins have been expressed in these cells. The E2 protein has been also obtained in the mammary gland of genetically modified organisms at high levels with protective capacity (Sánchez et al. 2014). However, this expression system is not technically feasible and it takes too long to generate a transgenic animal able to produce the E2 protein in the mammary gland. It had been also reported in the year 2008 the use of the PK-15 cell line as an expression system for production of E2. In this case, the results achieved in terms of high levels of expression and the ability of this antigen to confer protection against a viral challenge, suggest that the protein E2 produced in mammalian cells could constitute an efficient vaccine candidate for prevention and eradication of the CSF (Sánchez et al. 2008). On the other hand, a transgenic mammalian cell line has recently been established (based on the baby hamster kidney cells, BHK-21) which expresses stably the E2 protein. Vaccine formulations with the protein derived from this system confer protection against CSFV in a viral confrontation (Hua et al. 2014). However, with none of these antigens were possible to generate an early response in immunized pigs.

For that reason, recently we proposed a new vaccine candidate against CSFV, which is based on viral E2 glycoprotein fused to the extracellular domain of the porcine CD154 protein (Toledo et al. 2007; Pujol et al. 2015). This last protein has been used as a molecular adjuvant since several studies have claimed that it potentiates the immune system response (Xiang et al. 2001; Ramos et al. 2011). The signals triggered by the binding of this molecule to its receptor (CD154-CD40) are crucial for the proliferation and differentiation of the antigen-specific B cells, as well as for the change of isotype and maturation of the antibody’s affinity. All this is essential for the efficient generation of both memory B cells and long-lived plasma cells (Henn et al. 2001). The pig immunization using a unique dose of the chimeric protein E2-CD154 produced by HEK-293 cells growing in a serum-free suspension culture and the challenge experiment performed only 7 days after by infection with a high virulent CSF virus strain without clinical manifestations of the disease demonstrated the early protective capacity of this antigen in pigs (Suárez et al. 2017).

The HEK293 cell line is originally derived from Human Embryonic Kidney tissue and it has been extensively used as a recombinant expression system for heterologous proteins (Durocher and Butler 2009; Graham et al. 1977). Despite of its epithelial origin, the biochemical machinery of this cell line is able to perform most of the post-translational processing required to generate functional mature proteins. In addition, they are amenable for the rapid scale up of production processes, which can generate hundreds of milligrams of protein harvested in weeks (Henry and Durocher 2011; Loignon et al. 2008). Different strategies have been developed in order to increase the productivity and the cell density of these mammalian cell cultures which have included the improvements of expression vectors, culture media composition, cultivation process and host cell engineering (Dietmair et al. 2011). The most common attempts have focused on optimization of the culture media and the culture strategy such as batch, fed-batch, and perfusion (Liste-Calleja et al. 2014). Both culture conditions affect N-glycosylation which is often required for proper protein folding, protein–protein interactions, stability and optimal pharmacokinetics (Böhm et al. 2015). In the case of the E2 protein, glycosylation has demonstrated to be essential for the correct folding of the protein because the absence of these structures blocks the formation of the characteristic dimers of its structural conformation which consequently affects its biological functions (Tyborowska et al. 2007).

In this work, the growth and the expression levels of the recombinant HEK-293 cell line which produces the chimeric protein E2-CD154 in the supernatant of the suspension culture (HEK-293-E2-CD154) were characterized using four commercial culture media. The E2-CD154 proteins produced in 10L fermenters by these cells using the two media in which the cell performance was better were also characterized through the analysis of its *N*-glycan structures and its capacity to induce protective levels of neutralizing antibodies in mice vaccinated with experimental formulations of these proteins.

## Materials and methods

### Cell line and cell culture media

The cell line HEK293-E2-CD154 (Suárez et al. 2017): Human kidney embryonic cells (ATCC CRL-1573) adapted to stably transformed suspension growth that produce the E2-CD154 protein in the culture supernatant was used in all experiments. Three times a week, cell passaging was routinely performed in 125 mL plastic erlenmeyer flasks (Corning Inc., USA), seeding 15 mL of culture media with 0.3 × 10^6^ cells/mL. Flasks were shaken at 110 rpm on an orbital shaker (IKA, Germany) in an incubator (ASSAB Box 1715-17225, Sweden) with a temperature set at 37 °C in a humidified atmosphere with 5% of CO_2_. At each passaging, a sample of culture supernatant was taken to check the E2-CD154 protein expression. Cultures were grown up to 1 × 10^6^ cells/mL and then a new passaging was performed. ProS293CDM (Invitrogen, USA), CDM4HEK293 (HyClone, USA), SFM4HEK293 (HyClone, USA) and CPCHO (CIM, Cuba) culture media were used to growth cells.

### Cell growth assessment

HEK293 E2-CD154 cells were seeded in four replicates at 0.3 × 10^6^ cells/mL in a final volume of 30 mL using ProS293CDM, CDM4HEK293, SFM4HEK293 and CPCHO culture media. Cultures were carried out in 125 mL erlenmeyer flasks under the same culture conditions that those in maintenance passagings. Cell number from each culture was determined in duplicate every 24 h using a Neubauer emocytometer and a phase contrast microscope (ZEISS, Germany). Cell viability in the cultures was assessed using the Trypan blue dye exclusion method. Supernatant samples of each culture were taken every 24 h for quantification of E2-CD154 protein. The growth parameters of the cells in each medium were calculated using the following formulas:

$${\text{Cellular}}\;{\text{density:\;D}}\left( {\frac{{{\text{cells}}}}{{{\text{mL}}}}} \right) = \frac{{{\text{number}}\;{\text{of}}\;{\text{cells}}}}{4} \times {\text{dilution}}\;{\text{factor}} \times 10^{4}$$

$${\text{Cellular viability: V }}\left( \% \right) = \frac{\text{number of viable cells }}{{{\text{number of viable cells}} + {\text{number of dead cells}}}} \times 100$$

$${\text{Specific growth rate: }}\mu \left( {{\text{h}}^{ - 1} } \right) = \frac{{logN - logN_{0} }}{0.301} \times \left( {t - t_{0} } \right)$$

where N number of cells at time t, No: number of cells at time to, t: final time, to: initial time, 0.301: log2.

$${\text{Duplication time: td }}\left( {\text{h}} \right) = \frac{1}{\mu }$$

$${\text{Productivity: P }}\left( {{\text{E}}2 - {\text{CD}}154\,{\text{pg}}/{\text{cell}}} \right) = \frac{{{\text{E}}2 - {\text{CD}}154 {\text{pg in the supernatant}}}}{\text{number of viable cells}}.$$

### E2-CD154 production process in fermenter

Two fermentation process using the culture medium CDM4HEK293 and SFM4HEK293 (Batch 1 and Batch 2, respectively) were performed. These processes were carried out in a fermenter (BIOSTAT B Plus, Spain) with 8 L of culture effective volume in a 10 L glass reactor and a rotary filter (Sartorius, Germany) operated on a continuous infusion regimen at 37 °C of temperature, stirring at 150 rpm, pO_2_ = 20%, pH = 7,3 and an inoculum of 0.3 × 10^6^ cells/mL. The culture supernatants obtained in these conditions were concentrated using tangential ultrafiltration technology (100 kDa PESU cassette) until 150 mg of E2-CD154 protein/L. After that, the proteins were dialyzed against three volumes of 50 mmol/L of phosphate buffer and 0.3 mol/L sodium chloride, pH 7.2 ± 0.2. These materials were filtered by 0.2 µm to sterilize them.

### SDS-PAGE and Western blotting assays

E2-CD154 protein samples were separated by electrophoresis on SDS-PAGE gels at 10% as previously described (Sambrook et al. 1989), under reducing conditions (5% β-mercaptoethanol, 1% glycerol, 0.4% SDS and 12.5 mM Tris–HCl pH 6.6). In all cases, 10 μL of the sample was applied directly from the culture supernatant or 1 mL of precipitated sample. The specific E2CD154 protein concentration was not taken into account to apply the samples in the SDS-PAGE assays. Samples separated by SDS-PAGE were transferred to a Hybond-C nitrocellulose membrane using a semi-dry Mini Trans-Blot Electrophoretic Transfer Cell (Bio-Rad, USA). Western blot technique was performed using a monoclonal antibody against E2 protein conjugated to horseradish peroxidase diluted 1: 5000 (MAb-CBSSE2.3-HRP, CIGB Sancti Spíritus, Cuba).

### E2-CD154 quantification by sandwich ELISA

The E2-CD154 protein expression levels of the HEK293-E2-CD154 cell line were determined by a sandwich ELISA using the specific anti-E2-CSFV monoclonal antibodies 1G6 and CBSSE2.3-HRP (horseradish peroxidase-conjugated) (CIGB Sancti Spíritus, Cuba), as capture and detector antibodies, respectively. An E2-CD154 protein (Suárez et al. 2017) with a purity degree higher than 95% was used as standard.

### Molecular weight estimation of the E2-CD154 protein and prediction of its potential N-glycosylation sites

Masslynl and NetNGlyc programs were used to estimate the molecular weight and N-glycosylation sites of the E2-CD154 protein, respectively. The NetNGlyc program was also used to predict which of these potential sites are effectively N-glycosylated.

### N-Glycosylation profile from E2-CD154 proteins produced by recombinant HEK293 cells

The E2-CD154 proteins obtained from both fermenter processes were denatured at 70 °C for 10 min in 0.1% SDS, 5% β-mercaptoethanol and after cooled to room temperature. Nonidet P-40 (NP-40) was added to a final concentration of 1% before the PNGase F addition. Digestions were carried out using 5U of PNGase F per µg of glycoprotein at 37 °C during 2 h. The digestion results were visualized in a 10% SDS-PAGE gel under reducing conditions. *N*-Glycans derived from the PNGase F deglycosylation reactions were derivatized with 2-aminobenzamide (2AB) by a reductive amination reaction. The 2AB derivatives obtained were then analyzed by high-resolution liquid chromatography in normal phase (NP-HPLC) (Montesino et al. 2008).

### Oligosaccharides identification using the lectin specific binding

The ‘‘DIG Glycans Differentiation Kit’’ (Roche, Germany) and the Concanavalin A were used to identify specific E2-CD154-attached carbohydrate structures following the manufacturer’s instructions. Lectins specificities included in the assay are the followings: Concanavalin A recognizes terminal glucosamines and mannoses, *Galanthusnivalis agglutinin* (GNA) recognizes terminal mannoses and α(1-3), α(1-6) or α(1-2) linked to mannose, *Sambucusnigra agglutinin* (SNA) recognizes sialic acid linked α(2-6) to galactose and *Phaseolus vulgaris fitohemaglutinina* (PHA) recognizes β(1-4) linked galactoses.

### Immunogenicity assay in mice

The immunogenicity induced by the chimeric proteins E2-CD154.1 and E2-CD154.2 (Batch 1 and 2, respectively) was evaluated using Balb/C female mice eight to 10 weeks old and weighing 18–20 g obtained from the Center for the production of Laboratory Animals (CENPALAB, Havana, Cuba). The trial was conducted in the Animal House at CIGB. Mice were maintained in cages (× for each one) under 12:12 h light/dark regimen and fed with a pellet diet (produced by CENPALAB, Havana, Cuba) and water ad libitum. The sampling exercise and all procedures were carried out in accordance with the Guide for the Care and Use of Laboratory Animals. Mice were randomly assigned to 3 experimental groups with 10 mice each one. Groups 1 and 2 were immunized with 12.5 μg/mL of the E2-CD154.1 and E2-CD154.2 proteins, respectively. Both E2-CD154 proteins were experimentally formulated in Montanide ISA50TM V2 (SEPPIC) using a 60/40 proportion of aqueous/oil phase. “Water in oil” emulsions were obtained using an Ultra-Turrax T25 basic homogenizer (IKA Works Inc.). Group 3 was immunized with PBS1X in the same “Water in oil” emulsion. In all cases, 100 µL of final preparations was administered intraperitoneally in each mouse. Mice were immunized at days 0 and 21 and serum samples were taken on days 0 and 28 to measure the neutralizing antibody responses (NAb).

### Neutralizing antibody detection

Serum samples were screened for their capacity to neutralize the cell culture adapted Margarita strain of the CSFV from the National Center for Animal and Plant Health (CENSA, Mayabeque, Cuba) using the Neutralizing Peroxidase Linked Assay, NPLA (Terpstra et al. 1984). The assay was revealed with theanti E2 Mab CBSSE2.3 (CIGB-SS, Cuba) conjugated to horseradish peroxidase followed by DAB substrate. The presence of viral replication was determined by visual inspection at the optical microscope. The last serum dilution without any signal of virus replication was considered as the neutralizing titer.

### Statistics

Cell density and viability of cultures are expressed as the average of two independent counts for each sample from at least two biological replicates of each different culture condition. The error bars represent the standard deviations of the biological replicates. All data were compared by Ordinary one-way ANOVA and Tukey’s multiple comparisons Test using the statistical software Graph Pad Prismv.6.0 (GraphPad, USA).

## Results

### HEK293-E2-CD154 cell growth profiles

**Fig. 1:**
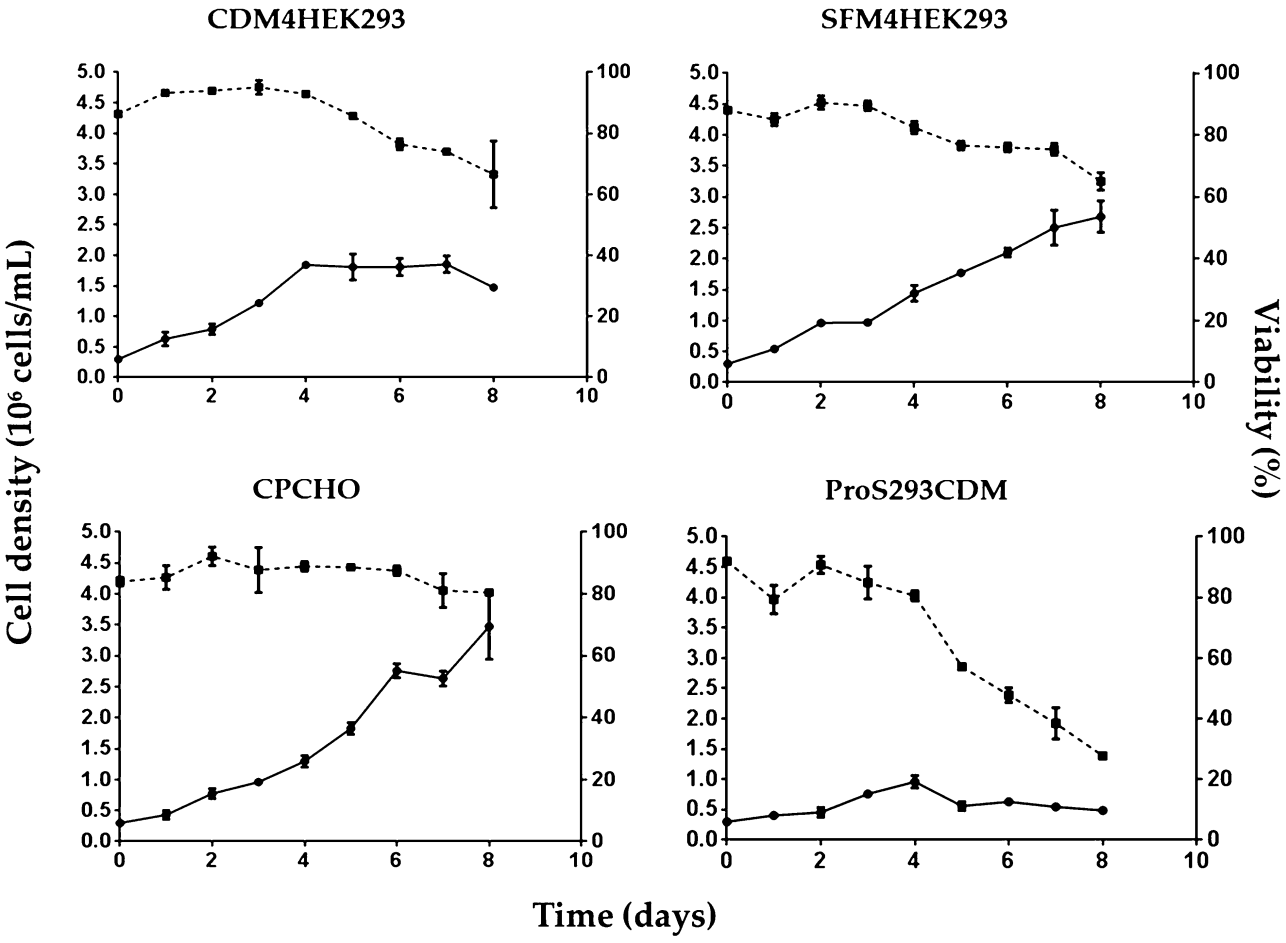
Cell density (circle-solid line) and viability (square-dashed line) profiles of HEK-293-E2-CD154 cell cultures with the four commercial media evaluated. **a** CDM4HEK293 culture media, **b** SFM4HEK293 culture media, **c** CPCHO culture media, **d** ProS293CDM. Each point represents the average from four determinations (two cell counts from two biological replicates). Error bars represent standard deviations

The growth kinetic of HEK-293-E2-CD154 cells with the four commercial culture media shows better cell growth in CPCHO, SFM4HEK293 and CDM4HEK293 media than the cell growing in Pro293S-CDM medium (Fig. 1). The maximum cell densities (X_max_) reached were 3.47 × 10^6^ cells/mL, 2.68 × 10^6^ cells/mL, 1.85 × 10^6^ cells/mL and 0.96 × 10^6^ cells/mL for each medium, respectively. On the other hand, the cell viability was consistently higher than 85% the first 5 days of culture when CPCHO and CDM4HEK293 media were used. In the case of cell cultures using SFM4HEK293 and Pro293S-CDM media, the cell viability was lower than 80% after 5 days of cell culture. Maximum cell densities (X_max_), maximum specific growth rates (µ_max_) and cell doubling times (t_d_) estimated in these assays for each culture medium are summarized in Table 1.

**Table 1:** Kinetic parameters of HEK293 E2-CD154 cells growing in different culture media

Kinetic parameters	Culture media			
	Pro293SCDM	CDM4HEK293	SFM4HEK293	CPCHO
X_max_ (× 10^6^ cell/mL)	0.96 ± 0.098	1.85 ± 0.13	2.68 ± 0.25	3.47 ± 0.53
µ_max_ (× 10^−2^ h^−1^)	1.61 ± 0.10	4.41 ± 0.06	3.36 ± 0.04	1.79 ± 0.02
t_d_ (h)	62.8 ± 10.8	23.4 ± 5.7	29.8 ± 2.1	58.2 ± 17.0

Each value represents the average from four determinations ± standard deviation

*X*_*max*_, maximum cell density; *µ*_*max*_, maximum specific growth rate; *t*_*d*_, cell doubling time

The highest µ_max_ values were obtained using CDM4HEK293 (0.044 h^−1^) and SFM4HEK293 (0.033 h^−1^) culture media which correspond to cell doubling times of 23 and 29 h, respectively. The µ_max_ values for cells growing in CPCHO and Pro293S-CDM culture media were about the half of those obtained with the previous media (0.018 h^−1^ and 0.016 h^−1^ respectively) while the cell doubling times in these media were substantially longer. In addition, the cell cultures with CDM4HEK293 and ProS293CDM media were kept during 8 days and the cultures using CPCHO and SFM4HEK293 media were performed for 10 days.

**Fig. 2:**
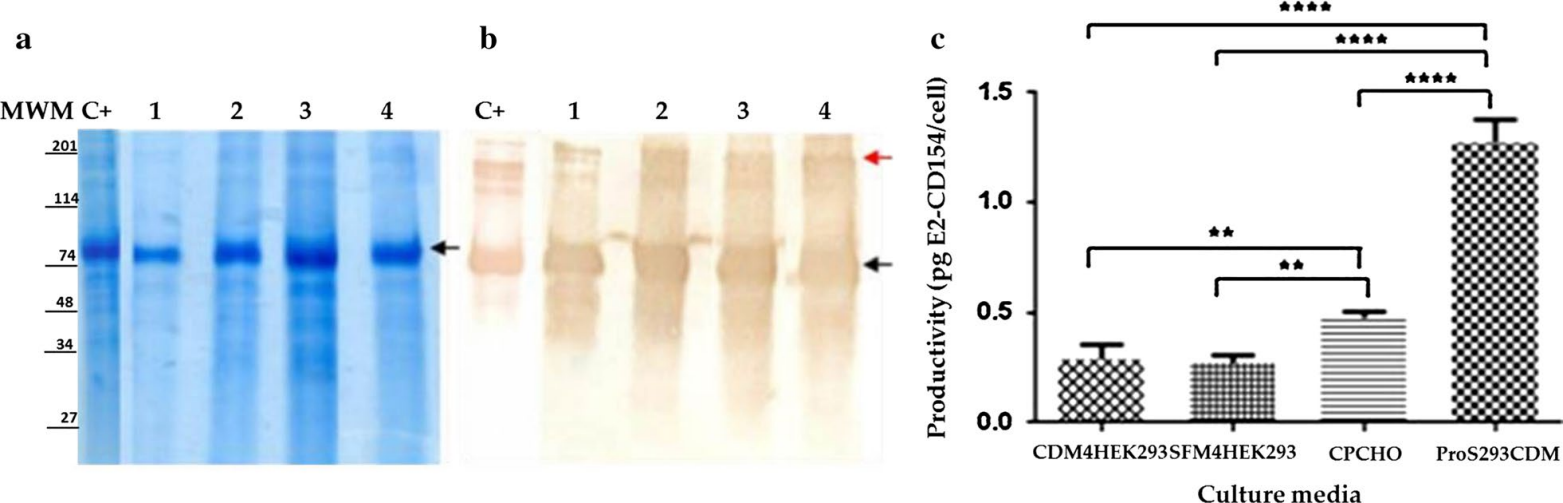
Analysis of the E2-CD154 proteins produced in the culture supernatants of HEK-293-E2-CD154 cells growing in the four different commercial media **a** SDS-PAGE 10% under reducing conditions, **b** Western blotting using MAb2.3HRP monoclonal antibody 1:5000. MWM: Molecular weight marker; C+: Positive control of E2-CD154 (Suárez et al. 2017); 1: Pro293SCDM medium; 2: CDM4HEK293 medium; 3: SFM4HEK293 medium; 4: CPCHO medium. In all cases, 10 µL of each culture supernatant from HEK-293-E2-CD154 cells were applied. **c** Productivity of HEK-293-E2-CD154 cells in terms of E2-CD154 protein pg per cell, which was calculated from four replicates in all cases. Error bars indicate the standard deviations. Asterisks mean statistical significance (**p < 0.005) and ***p < 0.0001)

The analysis of the culture supernatants of the HEK293-E2-CD154 harvested at the end of the kinetics study in different culture media was carried out by SDS-PAGE under reducing condition. In all cases, intense bands at an approximate size between 74 and 114 kDa according to the molecular weight standard used (Fig. 2a) were shown. This result corresponds to the expected size for the monomeric conformation of the glycoprotein E2 CD154. The Western blotting assay corroborated what was observed in the SDS-PAGE, showing immunoreactive bands at the same size. In addition, bands were immuno identified above 200 kDa corresponding to the estimated size for the dimers and other high molecular weight aggregates typical for the glycoprotein E2-CD154 (Suárez et al. 2017) (Fig. 2b).

The highest productivity of this cell line in terms of the E2-CD154 protein quantity expressed per cell was obtained when the cells were grown in the Pro293S-CDM media. This productivity was statistically significant respect to the E2-CD154 protein expressed by the cells growing in CPCHO, CDM4HEK293 and SFM4HEK293 culture media. Likewise, the quantity of E2-CD154 protein expressed by cells growing in CPCHO was statistically higher than the expression in CDM4HEK293 and SFM4HEK293 while there were no significant statistically differences between E2-CD154 expression in CDM4HEK293 and SFM4HEK293 culture media (Fig. 2c).

### Analysis of the E2-CD154 protein produced in 10 L fermenters

**Fig. 3:**
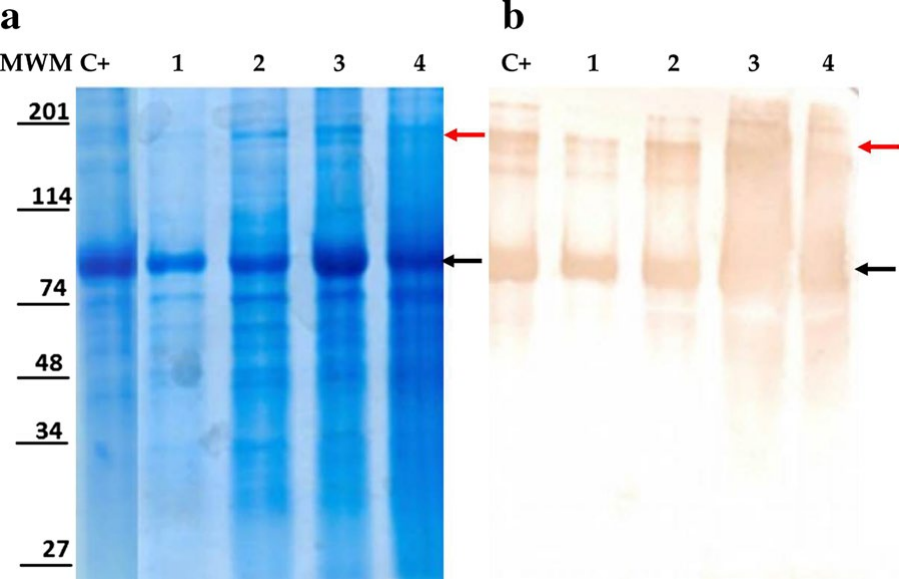
Analysis of the E2-CD154.1 and E2-CD154.2 proteins obtained in the experimental production processes in a 10 L fermenter **a** SDS-PAGE 10% under reducing conditions, **b** Western blotting using MAb2.3HRP monoclonal antibody 1:5000. MWM: Molecular weight marker; C+: Positive control of E2-CD154 (Suárez et al. 2017); 1 and 2: 10 µL of the batch 1 and batch 2 supernatants respectively applied directly. 3 and 4: 1 mL of the batch 1 and batch 2 supernatants respectively concentrated by precipitation. The black arrows indicate the probable E2-CD154 monomer and the red arrows pointed the putative E2-CD154 dimers and aggregates with high molecular weight

The SDS-PAGE analysis under reducing conditions of the E2-CD154.1 and E2-CD154.2 proteins produced in batch 1 y batch 2 of a 10 L fermenter respectively demonstrated that both proteins migrated as a wide band between 66 and 97 kDa according to the molecular weight marker used (Fig. 3). This result corresponds to the estimated size for the monomeric form of the protein that should be 70 kDa. In addition, bands with sizes greater than 200 kDa that could correspond with dimers and protein aggregates of higher molecular weight were observed in the Western blotting assay.

### Characterization of the N-linked oligosaccharides of the E2-CD154 protein produced in 10 L fermenters

**Fig. 4:**
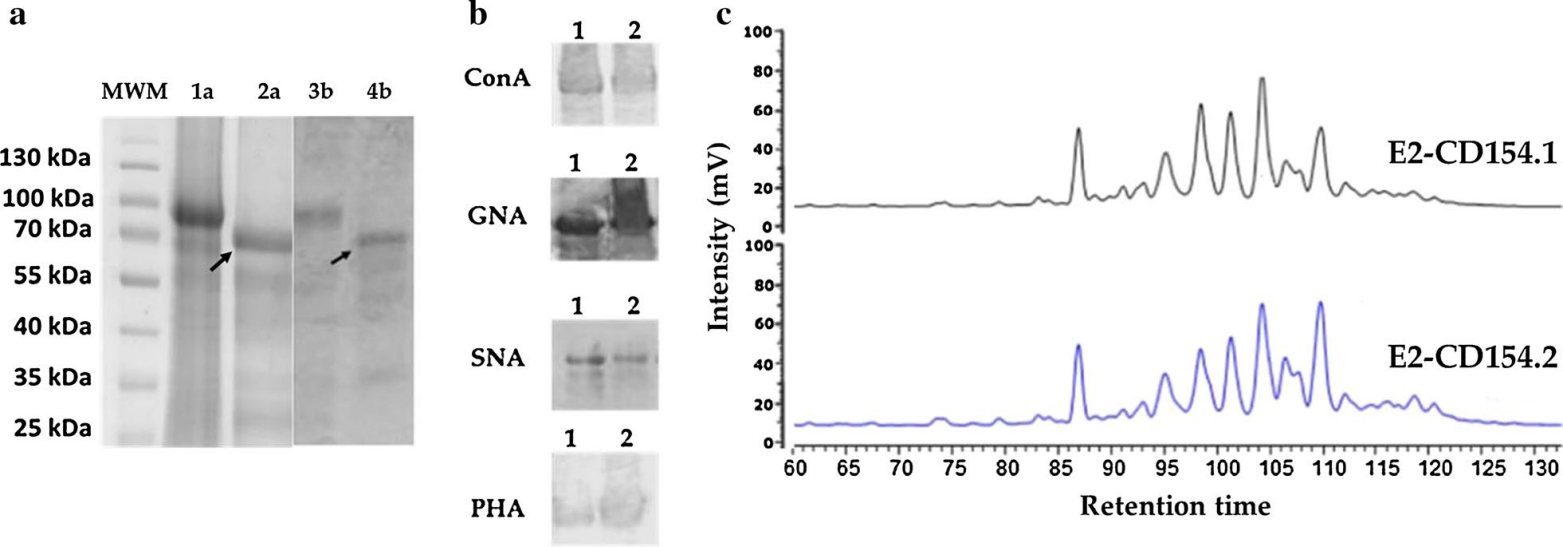
Characterization of the *N*-glycans linked to the E2-CD154 proteins. **a** 10% SDS-PAGE under reducing conditions. MWM: Molecular weight marker; 1a and 1b: E2-CD154.1 and E2-CD154.2 proteins expressed by HEK293 cells in fermenter respectively; 2a and 2b: E2-CD154.1 and E2-CD154.2 after PNGase F treatment, respectively. The arrows indicate the putative non-glycosylated proteins. **b** Results of lectin specific assays to detect the quality of *N*-glycans bound to both E2-CD154 proteins, 1: E2-CD154.1; 2: E2-CD154.2. **c** NP-HPLC chromatograms of 2AB derivatives from E2-CD154N-glycan

The SDS-PAGE analysis and the Western blot of E2-CD154.1 and E2-CD154.2 proteins after the N-deglycosylation assay performed by a PNGase F treatment demonstrated the presence of N-glycosylation in the proteins because they showed an incremented electrophoretic migration after this enzymatic digestion (Fig. 4a) The theoretical molecular weight of E2-CD154 chimeric protein is 70 kDa which is agree with the size observed for both proteins after treatment. On the other hand, the increased size observed for both proteins before treatment is agree with the presence of glucans in the seven potential N-glysosylation sites present in the protein, six in the E2 glycoprotein and one in the CD154 molecule.

Lectin-binding assay revealed the presence of oligomannoside, hybrid and complex *N*-glycans attached to both E2-CD154 proteins. Additionally, the presence of α(2-6) linked sialic acid was also confirmed for both proteins by positive SNA lectin recognition (Fig. 4b).

The NP-HPLC profiles from the *N*-glycans obtained from the PNGase F deglycosylation reactions of the two E2-CD154 preparations were very similar, which point the reproducibility and consistency of the E2-CD154 protein expressed by the HEK293 cells independently of the culture media used (Fig. 4c). Both chromatograms show the same fractions with small differences in their proportions. There are also in the profiles many wide peaks which suggest the co-elution of more than one structure of *N* glycan in the same fraction. This behavior is characteristic of N-glycosylation profiles where *N*-glycans with different sialylation degree coexist.

### Immunogenicity evaluation of two E2-CD154 experimental preparations

**Fig. 5:**
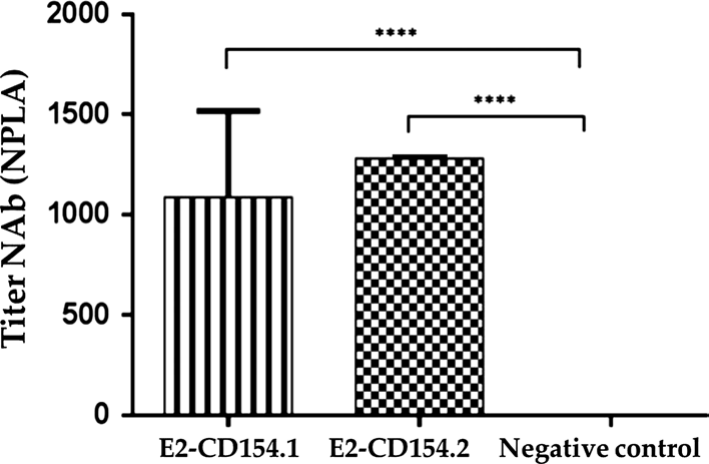
Antibody titers measured by NPLA 7 days after booster in the immunization experiment in mice. Data are expressed as the reciprocal of the average of antibody titers in each group. Neutralizing antibodies (NAb) were measured by the serum ability of each animal to neutralize 100 TCID50 of CSFV produced in 500 PK-15 cells. Asterisks represent statistical significant differences (p < 0.0001, Tukey’s multiple comparisons test). Standard deviations are represented

The E2-CD154.1 and E2-CD154.2 proteins demonstrated the same immunogenic capacity when mice were vaccinated with 12.5 μg/mL of each antigen as shown by the NPLA titers 7 days after the second immunization (Fig. 5). These neutralizing specific immune responses were statistically different (p < 0.0001) from the response in the control group vaccinated with placebo in which any specific neutralizing antibodies against E2-CSFV protein were detected. In addition, no significant statistical differences were observed between the responses of two E2-CD154 preparations tested, being in both cases the neutralizing antibody levels higher than the protective values reported.

## Discussion

The protein production using biotechnological approaches has a great impact for the vaccine industry worldwide. However, the selection of the suitable expression system is a main factor which determines the obtaining of these molecules in their active biologic forms. When mammalian cells as HEK293 are selected to establish a large-scale production process for a protein requiring N glycosylation as posttranslational modification as is the E2-CD154 case, it is important to choose the best culture medium to growth the cell line for obtaining the highest expression of that protein (Liste-Calleja et al. 2014; Hunter et al. 2019). According to our results, even though cellular productivity of E2-CD154 protein obtained in ProS293CDM culture medium was the highest, cell density and viability obtained during the growth kinetic study showed the lowest values in this culture medium when compared to the other media, suggesting that ProS293CDM medium is not adequate to growth our transformed HEK-293 cell line. These results are agreed with other authors (Geisse et al. 2005), wherein the HEK-293 cell density in that medium did not reach one million of cells per mL. However, considering the high productivity achieved with this culture medium, an optimization study to improve the cell density and viability could be performed using suitable additives (Liste-Calleja et al. 2014).

On the other hand, the cellular productivity of the E2-CD154 protein obtained using CPCHO medium showed statistically significant differences with respect to productivity achieved when cells were grown in the CDM4HEK293 and SFM4HEK293 culture media which are recommended for the HEK-293 growth. This result demonstrates that the culture medium CPCHO despite being a medium designed for culturing CHO cells could be used to growth the transformed HEK-293 cell line. Furthermore, the best cell density around 3.48 × 10^6^ cells/mL during the growth assay was obtained with this medium. There were not significant differences between E2-CD154 productivities achieved when cells were grown in SFM4HEK293 and CDM4HEK293 culture media. However, they were lower than those obtained in CPCHO and ProS293SCM media, as mentioned above. In general, our productivity results were lower than those reported by other authors with the same media, which may be due to that our productivities were calculated after 24 h of culture compared to values calculated after 48 and 72 h of culture (do Amaral et al. 2016). Meanwhile, the growth kinetic assay showed cell densities for both culture media over a million of cells per mL, which exceed the values achieved when the culture medium ProS293CDM was used to growth the cell line.

According to the results of this study, culture media are a key factor that influences most of the kinetic parameters. This may be mainly due to the specific composition of each culture medium (Thermo Scientific Hyclone Cell Culture 2011 Product Manual). For these reasons the selection of the appropriate medium is of great importance for the development and definition of the recombinant protein productive processes. From the economic point of view, the selection of the culture medium is also an important factor for the productive processes. The commercial media CDM4HEK293, SFM4HEK293 and ProS293CDM used in this study cost 79.36, 44 and 95 USD per liter, respectively (GEHalthcare Life Sciences and LONZA catalogs). However, the CPCHO medium has a cost of 3.7 USD per liter, which represents a saving of more than 90%. Taking into account these data, and the results obtained in this work, it is demonstrated that the use of the CPCHO medium represents a promising option for scaling the culture of HEK293-E2-CD154 cells with high feasibility economical.

Despite of good results described before with CPCHO medium, if μ_max_ and t_d_ showed in the Table 1 are taken in consideration, it can be noticed that the best specific growth rates were obtained when the cells were grown in media SFM4HEK293 and CDM4HEK293.

Because of this, these two culture media were used to produce two experimental production process in a 10L fermenter with the aim to analyze conformations and glycosylation pattern of the protein E2-CD154 expressed by HEK293 cells in both culture media. According to SDS-PAGE and Western blotting analysis of the E2-CD154 protein produced by this cell line in the culture supernatant of the fermenter batches 1 and 2, the monomeric and homodimeric conformations of the protein were present and they were recognized by a monoclonal antibody that recognizes the E2protein in reducing conditions (Sánchez et al. 2014). These results demonstrate that the structure of the E2-CD154 protein expressed by HEK 293 cells is not affected by differences in culture media used to growth cells. In the same way, profiles of N-linked oligosaccharides to E2-CD154 protein obtained in both culture media were highly similar with the same neutral species with low degree of polymerization and the same charged large size oligosaccharides. These results indicate that the N-glycosylation developed by the biosynthetic machinery of the HEK293-E2-CD154 transformed cells is consistent and independent of the culture medium used. This is an important issue to take into account for the development of a vaccine antigen production process based on the chimeric protein E2-CD154, since it has been demonstrate that glycosylation plays an essential role in the correct folding of the E2 protein and in the formation of homodimers that are determinants in their biological function (Tyborowska et al. 2007).

Once it was demonstrated that the CDM4HEK293 and SFM4HEK293 culture media did not influence the structural conformations of the E2-CD154 protein neither its N-glycosylation profile, the immunogenicity induced by these antigens in mice was evaluated using vaccine experimental formulations with E2-CD154.1 and E2-CD154.2 proteins. The results of this test demonstrated that both proteins induce neutralizing antibodies (NAb) titers greater than 1/1280. These levels of NAb are higher than those required to guarantee protection against a lethal challenge with a high virulent strain of CSFV. For live attenuated vaccines, titers ≥ 1/32 is reported protective (Terpstra and Wensvoort 1988). However, for a subunit vaccine based on E2 protein produced in insect cells, titers of AcN ≥ 1/50 are considered protective (Bouma et al.^1999^). These differences in the titer threshold to obtain protection could be due to the fact that the effector mechanisms of the immune response induced by live vaccines and by subunit vaccines are different (Postel et al. 2018). Based on this information, it can be stated that the glycoprotein E2-CD154 produced in the culture supernatant of the HEK293-E2-CD154 cells induces NAb titers exceeding in more than 20 times the antibody levels proposed to guarantee protection against viremia and clinical signs of the disease. The fact that the culture media do not affect the NAb response generated in vaccinated animals with any protein is agree with the result that the conformation neither the N-linked glycans to the E2-CD154 protein are affected by the culture conditions in which the protein is produced.

In summary, the results obtained in the present work suggest that the CDM4HEK293 and SFM4HEK293 culture media which are recommended for HEK-293 growth are the best choice to growth the cell clone expressing the E2-CD154 protein. The results also indicate that the HEK293-E2-CD154 cells are able of producing the E2-CD154 protein in the culture supernatant with similar conformational and immunogenic characteristics independently of the media in which they are cultivated which demonstrates the high reproducibility and consistency among protein batches produced by HEK-293 cells even in different culture conditions. It constitutes a very important characteristic to use this cell line in the production process of this vaccine antigen.

## Data Availability

It is stated that all data and material necessary is presented in the main paper.
